# Multiple Bladder Diverticula Presenting in an 82-Year-Old Congolese Male

**DOI:** 10.1155/2022/9295861

**Published:** 2022-06-10

**Authors:** Jean de Dieu Tumusifu Manegabe, Daniel Safari Nteranya, Ghislain Maheshe Balemba, Fabrice Cikomola Gulimwentuga, Paul Budema Munguakonkwa, Paterne Safari Mudekereza, Bijou Safi Matabaro, Alliance Wani Bisimwa, Franck Masumbuko Mukamba, Georges Kuyigwa Toha, Kizito Mutuga Centwali, Costa Sudi Musilimu, Zénon Kuyigwa, Alain Kabakuli Namugusha, Wani Mulumekandi Rugarabura, Léon-Emmanuel Mubenga Mukengeshai

**Affiliations:** ^1^Faculty of Medicine, Catholic University of Bukavu, Bukavu, Democratic Republic of the Congo; ^2^Surgery Department, Provincial General Hospital of Bukavu, Bukavu, Democratic Republic of the Congo; ^3^Surgery Department, University Clinics of Bukavu, Official University of Bukavu, Bukavu, Democratic Republic of the Congo; ^4^Department of Radiology, Provincial General Hospital of Bukavu, Democratic Republic of the Congo; ^5^Nursing Sciences Section, Superior Institute of Medical Techniques of Bukavu, Bukavu, Democratic Republic of the Congo

## Abstract

Bladder diverticulum is a congenital malformation characterized by the outpouching of the bladder following an obstruction of urine flux. We present a case of 82-year-old Congolese male patient presented at our facility with a poor urinary stream and lower abdominal pain. A distended abdomen was found on physical examination while the external genitalia were normal. All blood laboratory values were found to be within normal ranges. The patient's urine analysis revealed an uncountable number of white blood cells. Ultrasonography revealed multiple diverticula in the right posterolateral and posterior wall. An ultrasound of the abdomen revealed numerous bladder diverticula in the bladder's left posterolateral and posterior aspects, mild right-sided hydronephrosis, and severe left hydronephrosis with a thinned-out cortex. Both ureters were normal. A computed tomographic (CT) scan of the abdomen confirmed the diagnosis. The patient underwent an open laparotomy which allowed complete ablation of the diverticula followed by bladder wall repair. A one-week course of antibiotics was prescribed, and the patient was discharged fully recovered with no immediate complications. Although bladder diverticula are a congenital malformation, the presence of multiple diverticula suggests that the condition is acquired. In elderly patients, open laparotomy combined with intravenous antibiotics yields positive results.

## 1. Introduction

Generally defined as a herniation of the mucosa that lacks a muscle layer, bladder diverticulum results in contractility loss and urine stasis in the diverticulum [1–3]. It results from a defect in the bladder muscle development during the embryonic stage. Congenital bladder diverticula have been reported in 1.7 percent of children, with a peak in children under the age of 10 [2–4]. Generally, they are located superolateral to the ureteral orifice, close to the ureterovesical junction [1]. Bladder diverticula are typically discovered during an investigation for lower urinary tract symptoms (LUTS), hematuria, infection, stone formation, or malignant neoplastic change [5, 6]. We present a case of an 82-year-old adult with multiple bladder diverticula diagnosed at his advanced age.

## 2. Case Presentation

Over the last year, an 82-year-old Congolese male patient presented with a poor urinary stream and lower abdominal pain. On examination, the patient's abdomen was distended with no noticeable mass. He had a grossly distended bladder extending from the hypogastrium up to the right hypochondrium. The external genitalia were normal. All blood laboratory values were found to be within normal ranges. The patient's urine analysis revealed an uncountable number of white blood cells. Ultrasonography revealed diverticula in the right posterolateral and posterior wall. An ultrasound of the abdomen revealed numerous bladder diverticula in the bladder's left posterolateral and posterior aspects, mild right-sided hydronephrosis, and severe left hydronephrosis with a thinned-out cortex. Both ureters were normal.

**Figure 1:**
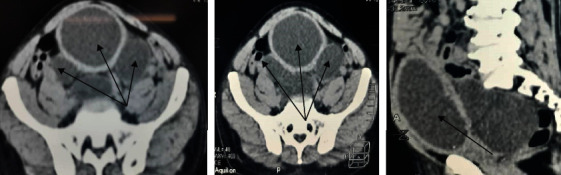
Computed tomographic (CT) scan of the abdomen and pelvis showing multiple bladder diverticula (black arrow).

**Figure 2:**
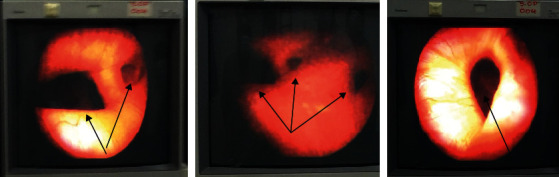
Cystoscopic image of multiple diverticula (black arrow).

**Figure 3:**
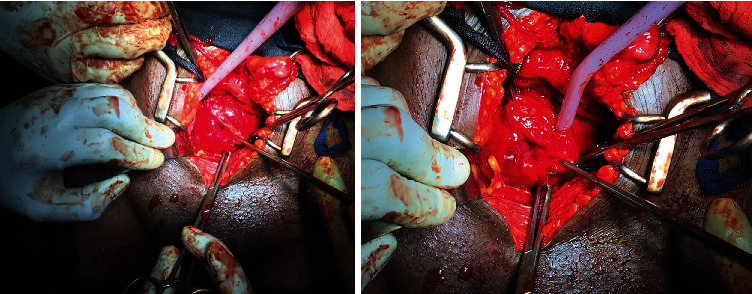
Open surgical repair of multiple bladder diverticula.

A computed tomographic (CT) scan of the abdomen and pelvis revealed a well-distended bladder with a significantly thickened and irregular wall measuring up to 1 cm with the presence of diverticula at its left lower posterior aspect (Figure 1). An International Prostate Symptom Score (IPSS) of 4 was obtained for the evaluation of bladder outlet obstruction, indicating that the symptoms are mild. Cystoscopy revealed a normal urethra with multiple wide-mouthed diverticula arising from the bladder's left posterolateral and posterior walls, but the left ureteric orifice was not visible (Figure 2). Prior to surgery, the urinoculture conducted revealed the presence of *Citrobacter freundii* sensible to meropenem and amikacin. For seven days, an intravenous antibiotic treatment of 1 g of meropenem thrice daily was administered. The patient underwent transvesical diverticulectomy, cystostomy, and abdominal washing drainage (Figure 3). Following a supine position, anesthesia, disinfection, and towel lying, we opened the bladder through the Pfannenstiel abdominal incision into the anterior space of the bladder, turned the diverticulum inside out, peeled off the diverticulum completely, sewed it up, and made a fistula at the same time. Cystotomy revealed that the largest size of the right diverticulum was approximately 22 × 12 × 20 cm^3^. About 700 mL of clear urine had been collected through the diverticulum's ostium. Following surgery, the patient experienced an 80% improvement in his symptoms while on intravenous antibiotics for one week, he was discharged on oral antibiotics for a total of three weeks. An abdominal ultrasound was performed 48 hours later as a follow-up after the surgery and revealed no evidence of diverticulum. We were unable to perform uroflowmetry due to a technical issue: our sole uroflowmeter being out of order.

## 3. Discussion

Bladder diverticula are a relatively uncommon clinical entity in both pediatric and adult populations. They are often referred to as “hernias of the bladder mucosa through the muscular fibers of the bladder wall,” resulting in a thin-walled structure connected to the bladder lumen [1]. They can be congenital or acquired. Congenital bladder diverticula occur in the absence of obstruction to the bladder outlet, often associated with a smooth-walled bladder with no trabeculations on cystoscopy [1, 3], while acquired bladder diverticula are due to all factors leading to obstruction of the bladder outlet such as swollen prostate (prostate adenoma or adenocarcinoma) or urethral stenosis [3, 4, 7].

They are linked to genetic disorders such as Ehlers-Danlos (type 9) syndrome, Menkes kinky hair syndrome, cutis laxa syndrome (Sotos), and Williams-Beuren syndrome. They require genetic testing to determine the etiology of the disease [2, 4, 8].

Bladder diverticulum diagnosis is in the majority of cases incidental (they can be discovered while the patient is undergoing imaging exams indicated for lower urinary tract symptoms). Their true incidence is unknown but is reported to be 1.7 percent in the pediatric population [2, 9].

Although rarely diagnosed in childhood, congenital bladder diverticulum is discovered incidentally following urinary tract infections [10]. Few cases of antenatal diagnosis have been reported [11, 12]. Generally, due to the asymptomatic evolution of the disease, the diagnosis is made in adulthood. In adults, bladder diverticulum is often revealed by lower urinary tract symptoms following benign prostatic hypertrophy. The diagnosis is easy and can be confirmed by an abdominal ultrasound [6, 13]. To refine the diagnosis, further explorations are often required: urethrocystography and urodynamic screening. The abdominal CT scan is often performed especially when there is a suspicion of an intradiverticular mass or a malignant origin or complication [1, 14, 15].

Bladder diverticulum often evolves towards malignancy. Intradiverticular cancer often develops within the diverticula. The most reported histologic types are urothelial carcinoma and urethral squamous papilloma [7, 15–17].

Diverse therapeutic approaches have been developed for bladder diverticulum management. While open surgery is widely preferred by surgeons for diverse reasons, the laparoscopic and transurethral endoscopic approaches are little by little supplanting it [10, 14, 18].

Actually, the robotic surgery has revolutionized the management of bladder diverticula [19]. The robot-assisted transvesical diverticulectomy is accompanied by some advantages: quick localization of the diverticulum and orifices, direct access to the prostate when simultaneous desobstruction is necessary, and short catheterization time [20, 21].

Open diverticulectomy (intra- or extravesical) is beneficial in the case of concomitant prostatic enlargement, allowing simultaneous treatment of both entities. The extravesical approach is reserved for patients with large diverticula associated with peridiverticular adhesions or inflammation. The laparoscopic approach, which offers the benefits of minimally invasive surgery, may also be used [3, 4, 10, 14, 18]. Endoscopic fulguration offers comparatives and satisfactory results in the management of acquired diverticula larger than 4 cm according to Pacella et al. [22].

## 4. Conclusion

Multiple bladder diverticula are rare acquired malformations. They are generally associated with urine flow obstruction and often diagnosed incidentally in adulthood following lower tract urinary symptoms. Abdominal ultrasonography coupled with abdominal computed tomography plays a key role in the diagnosis, while open surgery is the best procedure for elder patients.

## Data Availability

All materials used in this study are available on request.

## References

[1] Garat J. M., Angerri O., Caffaratti J., Moscatiello P., Villavicencio H. (2007). Primary congenital bladder diverticula in children. *Urology*.

[2] Abou Zahr R., Chalhoub K., Ollaik F., Nohra J. (2018). Congenital bladder diverticulum in adults: a case report and review of the literature. *Case Reports in Urology*.

[3] Jiang S., Ren Q., Wang X. (2020). A huge bladder diverticulum in an elderly: a case report. *SAGE Open Medical Case Reports*.

[4] Chen J., Mao J., Ye L., Zong D., Qiao X. (2020). Multiple bladder diverticula with Williams-Beuren syndrome: a case report. *Translational Pediatrics*.

[5] Sengupta S., Basu S., Ghosh K., Sengupta S. (2021). Urethral squamous papilloma with multiple bladder diverticulum: a case report and literature review. *Urology Case Reports*.

[6] Iscaife A., Anjos G., Barbosa Neto C., Nahas W. C., Srougi M., Antunes A. A. (2018). The role of bladder diverticula in the prevalence of acute urinary retention in patients with BPH who are candidates to surgery. *International Brazilian Journal of Urology*.

[7] Fang C. W., Hsieh V. C. R., Huang S. K. H., Tsai I. J., Muo C. H., Wu S. C. (2019). A population-based cohort study examining the association of documented bladder diverticulum and bladder cancer risk in urology patients. *Plo S One*.

[8] Pradhan N., Shilawant J., Akkamahadevi C. H., Shivakumar K. S., Sundaresh D. C. (2020). Ehlers-Danlos syndrome with huge bladder diverticulum in pregnancy - A rare and interesting case report. *European Journal of Obstetrics & Gynecology and Reproductive Biology*.

[9] Blane C. E., Zerin J. M., Bloom D. A. (1994). Bladder diverticula in children. *Radiology*.

[10] Celebi S., Sander S., Kuzdan O. (2015). Current diagnosis and management of primary isolated bladder diverticula in children. *Journal of Pediatric Urology*.

[11] Gaudet R., Heim N., Merviel P. (1999). Prenatal diagnosis of a congenital bladder diverticulum. *Fetal diagnosis and therapy*.

[12] Filho L. G. F., Marani A. L., Brollo L. F., Ocké J. C., Kaminagakura F. G., Budib L. J. (2021). Prenatal hydronephrosis revealing a bladder diverticulum. *Urology Case Reports*.

[13] Lahham S., Gutierrez S. (2021). Diagnosis of bladder diverticula with point-of-care ultrasound. *Clinical Practice and Cases in Emergency Medicine*.

[14] Pacella M., Testino N., Mantica G., Valcalda M., Malinaric R., Terrone C. (2019). Transurethral endoscopic approach for large bladder diverticula: evaluation of a large series. *Archivio Italiano di Urologia e Andrologia*.

[15] Di Paolo P. L., Vargas H. A., Karlo C. A. (2015). Intra-diverticular bladder cancer: CT imaging features and their association with clinical outcomes. *Clinical imaging*.

[16] Voskuilen C. S., Seiler R., Rink M. (2020). Urothelial carcinoma in bladder diverticula: a multicenter analysis of characteristics and clinical outcomes. *European urology focus*.

[17] Whitefield B. (2010). Urinary bladder diverticulum and its association with malignancy: an anatomical study on cadavers. *Romanian Journal of Morphology and Embryology*.

[18] Thwaini A., McLeod A., Nambirajan T. (2008). Laparoscopic bladder diverticulectomy. *Journal of Laparoendoscopic & Advanced Surgical Techniques*.

[19] Preciado-Estrella D. A., Cortés-Raygoza P., Morales-Montor J. G., Pacheco-Gahbler C. (2018). Multiple bladder diverticula treated with robotic approach-assisted with cystoscopy. *Urology Annals*.

[20] Develtere D., Mazzone E., Berquin C. (2022). Transvesical approach in robot-assisted bladder diverticulectomy: surgical technique and outcome. *Journal of Endourology*.

[21] Cacciamani G., De Luyk N., De Marco V. (2018). Robotic bladder diverticulectomy: step-by-step extravesical posterior approach–technique and outcomes. *Scandinavian Journal of Urology*.

[22] Pacella M., Mantica G., Maffezzini M. (2018). Large bladder diverticula: a comparison between laparoscopic excision and endoscopic fulguration. *Scandinavian Journal of Urology*.

